# PARP1 inhibition radiosensitizes HNSCC cells deficient in homologous recombination by disabling the DNA replication fork elongation response

**DOI:** 10.18632/oncotarget.6947

**Published:** 2016-01-19

**Authors:** Stephanie Wurster, Fabian Hennes, Ann C. Parplys, Jasna I. Seelbach, Wael Y. Mansour, Alexandra Zielinski, Cordula Petersen, Till S. Clauditz, Adrian Münscher, Anna A. Friedl, Kerstin Borgmann

**Affiliations:** ^1^ Laboratory of Radiobiology and Experimental Radiooncology, Center of Oncology, University Medical Center Hamburg-Eppendorf, Hamburg, Germany; ^2^ Tumor Biology Department, National Cancer Institute, Cairo University, Cairo, Egypt; ^3^ Department of Radiotherapy and Radiooncology, University Medical Center Hamburg-Eppendorf, Hamburg, Germany; ^4^ Department of Pathology, University Medical Center Hamburg-Eppendorf, Hamburg, Germany; ^5^ Department of Otorhinolaryngology and Head and Neck Surgery, University Medical Center Hamburg-Eppendorf, Hamburg, Germany; ^6^ Department of Radiation Oncology, Ludwig Maximilians University, Munich, Germany; ^7^ Clinical Cooperation Group ‘Personalized Radiotherapy of Head and Neck Cancer’, Helmholtz Center Munich, Neuherberg, Germany

**Keywords:** homologous recombination, PARP1 inhibition, replication stress, ionizing radiation

## Abstract

There is a need to develop new, more efficient therapies for head and neck cancer (HNSCC) patients. It is currently unclear whether defects in DNA repair genes play a role in HNSCCs' resistance to therapy. PARP1 inhibitors (PARPi) were found to be “synthetic lethal” in cancers deficient in BRCA1/2 with impaired homologous recombination. Since tumors rarely have these particular mutations, there is considerable interest in finding alternative determinants of PARPi sensitivity. Effectiveness of combined irradiation and PARPi olaparib was evaluated in ten HNSCC cell lines, subdivided into HR-proficient and HR-deficient cell lines using a GFP-based reporter assay. Both groups were equally sensitive to PARPi alone. Combined treatment revealed stronger synergistic interactions in the HR-deficient group. Because HR is mainly active in S-Phase, replication processes were analyzed. A stronger impact of treatment on replication processes (*p* = 0.04) and an increased number of radial chromosomes (*p* = 0.003) were observed in the HR-deficient group. We could show that radiosensitization by inhibition of PARP1 strongly correlates with HR competence in a replication-dependent manner. Our observations indicate that PARP1 inhibitors are promising candidates for enhancing the therapeutic ratio achieved by radiotherapy via disabling DNA replication processes in HR-deficient HNSCCs.

## INTRODUCTION

Head and neck squamous cell carcinoma (HNSCC) is the sixth leading cause of cancer, accounting for roughly 4–6% of all new tumors diagnosed. Overall survival rates have not significantly changed over the past 30 years and for patients presenting with locally invasive stage III and IV, with only 30% long term survival. Radiotherapy has played an increasingly important role in improving the poor survival rate of patients. Current treatment regimens using a combination of radiotherapy and standard chemotherapeutic agents such as cisplatin, 5-Flourouracil and etoposide have proved to be effective; however, combined modality treatments often result in unacceptable levels of toxicity to the patient [1].

In search for suitable alternatives, considerable interest has recently focused on DNA repair pathways as potential targets for novel cancer treatments [2]. The poly(adenosine diphosphate ribose) polymerase (PARP) family of DNA end-binding nuclear proteins is involved in one such pathway of DNA repair [3] and inhibition of PARP1 (PARPi) has become a promising therapeutic approach for the treatment of certain types of cancers, especially breast and ovarian cancer. It plays a critical role in the base excision repair pathway (BER), and is a key factor in the repair of single-strand breaks. Notably, a recent study also showed a function of PARP1 in alternative end-joining processes [4]. The popularity of PARP inhibitors is based on the observation that PARP inhibitors could selectively kill homologous recombination (HR)-deficient cancer cells [5, 6], enabling a so-called “synthetic lethality” approach. The reason behind the sensitivity of HR-deficient cancer cells to PARP inhibition is thought to be the accumulation of single-stranded DNA breaks in the absence of PAR synthesis, leading to replication fork collapse and double-stranded breaks, which require HR factors for repair [7]. Apart from its direct involvement in repair processes, PARP1 activity has also recently been reported to play a direct role in the control of replication [8, 9]. These observations make the combination of PARPi with irradiation attractive. Ionizing irradiation produces base damage and single strand breaks that the replication fork encounters and tumor cells with a defective regulation of HR and/or replication control are selectively inactivated through perturbed replication processes.

Radiosensitization by PARPi has been observed in a variety of different tumor types in preclinical models [10–16] and there is evidence that the radiosensitization depends on the fraction of cells in S phase [17, 18]. However, in HNSCCs mutations in HR genes are rare and not all mutation carriers with familial breast cancer respond to PARPi [19]. On the other hand, it was recently shown that also sporadic breast tumors which fail to form RAD51 foci could be sensitized by PARPi treatment although they share no common mutations in HR genes [20]. Therefore, other processes affecting HR might be involved in the cellular response to PARPi, influencing the radiosensitization effect.

This idea is further supported by the observation that haploinsufficiencies in HR genes, such as PALB2 or BRCA1 are sufficient to disturb replication processes [21] whereas common HR functions remain unaffected [22]. Assuming that a deficiency in HR together with an increase in replication stress makes tumors susceptible to radiosensitization by PARPi we performed a study in ten HNSCC cell lines to explore its potential use in the clinic.

## RESULTS

### Differences in the HR capacity in HNSCC cells

**Figure 1:**
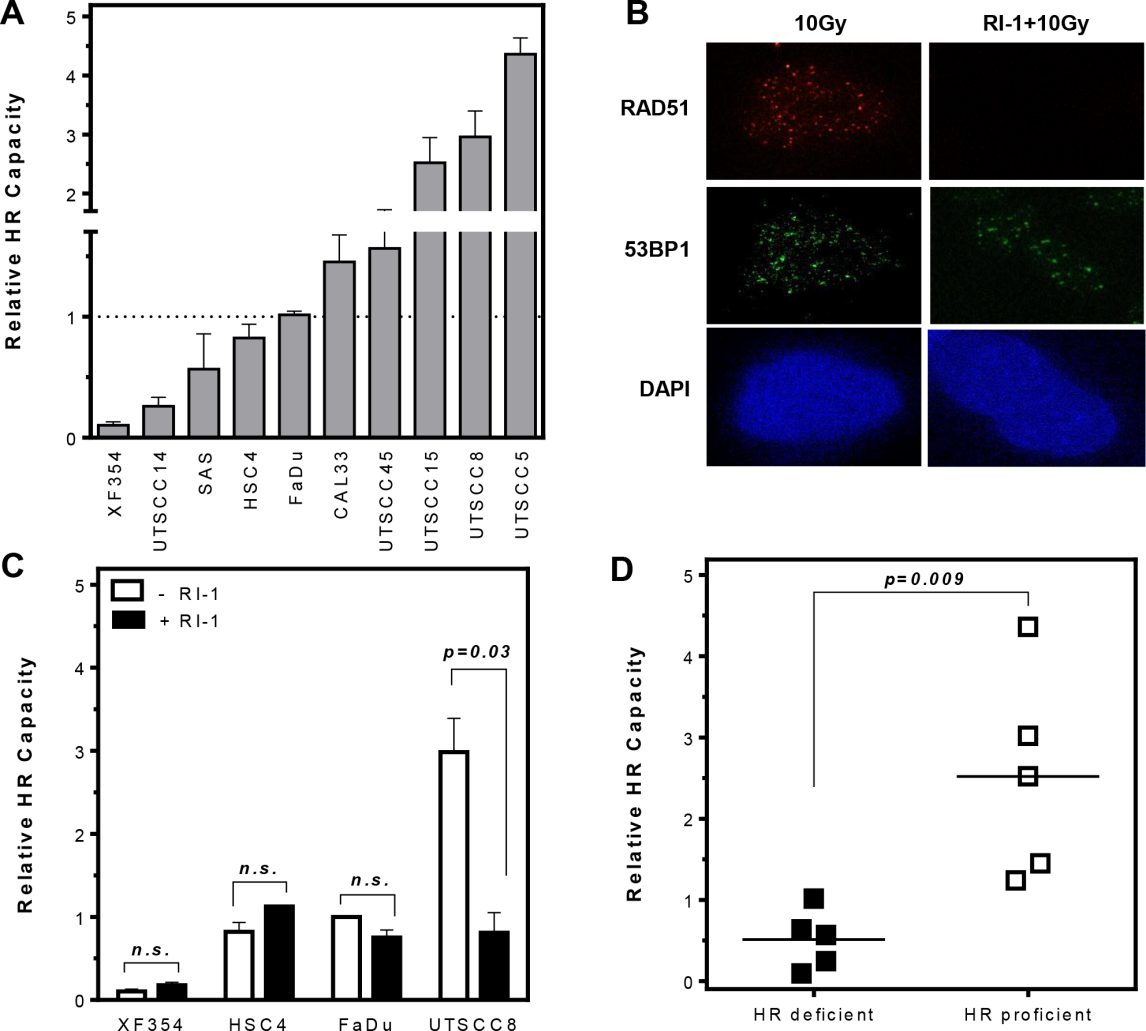
Variation in HR capacity allows discrimination of HR-proficient and HR-deficient HNSCC cell lines (**A**) HR capacities (relative to that of FaDu cells) were analyzed after transient transfection of an HR-specific GFP-reporter construct. (**B** and **C**) Inhibition of RAD51 by 20 μM RI-1 abolishes RAD51 foci formation and suppresses HR capacity only in HR-proficient cells (*p* = 0.03). (**D**) Classification of HNSCC cells into HR-proficient and HR-deficient cell lines (*p* = 0.009). Statistical analysis of at least three different experiments was performed using Student's *t*-test.

We have previously shown that breast cancer cell lines show differences in the HR capacity although they share no common mutations in BRCA1 or BRCA2 [23]. This also offers the opportunity to apply PARPi for the treatment of sporadic breast tumors to intensify tumor therapy. To evaluate if this observation is true for other tumor entities, enabling the use of specific PARPi for radiosensitization, HR capacity was analyzed in ten HNSCCs. Based on our observation that HR capacity measured by an HR-specific GFP based reporter assay show the same trend after stable as well as transient transfection [23] we performed only transient transfection. We could show that also HNSCC cells differ in their HR capacity analyzed after transient transfection of an HR-specific GFP-reporter construct (Figure 1A). All cell lines analyzed, except UTSCC5 cells, had a similar fraction of G1 phase cells, thus ruling out any confounding effects of cell cycle distribution on HR capacity (Figure S1A). To strengthen our observation, HR was inhibited by a RAD51-specific inhibitor RI-1 [24] which covalently binds to the surface of the RAD51 protein and prevents RAD51 oligomerization into filaments on DNA. Incubation with the RI-1 inhibitor showed a concentration-dependent decrease of HR capacity up to 30 μM (Figure S1B). Inhibition of RAD51 by 20 μM RI-1 completely abolished RAD51 foci formation (Figure 1B). However, HR capacity was only suppressed by RI-1 in the HR-proficient cell line UTSCC8 (Figure 1C and S1B) whereas the already HR-deficient cell lines, XF354, FaDu and HSC4 showed no change in HR capacity. To confirm the differences observed in HR capacity, cellular sensitivity after MMC treatment was determined in six out of ten HNSCC cell lines [25, 26]. As expected, MMC preferentially sensitized HR-deficient cells compared to HR-proficient cells (Supplementry Figure 1C). Based on their differences in HR capacity HNSCCs were grouped into HR-proficient and HR-deficient cell lines (*p* = 0.009). No difference in the expression patterns of relevant proteins such as BRCA1, PARP1 or CHK1 was observable between the two groups. Only RAD51 and FANCD2 seemed to be slightly over-expressed in most of the HR-deficient cell lines (Figure S1D).

### More effective radiosensitization by PARPi in HNSCCs with low HR capacity

**Figure 2:**
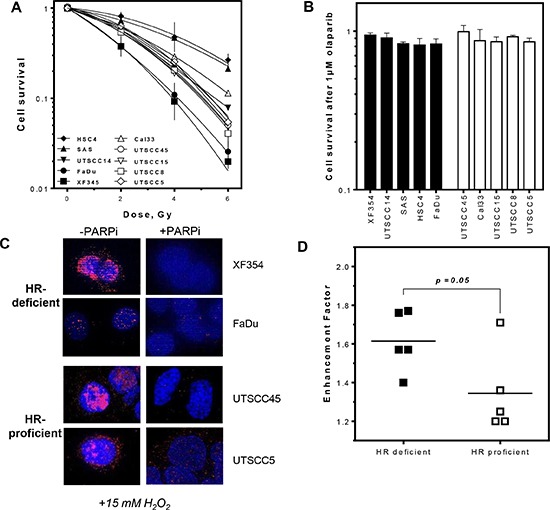
More effective radiosensitization by PARPi in HR-deficient compared to HR-proficient HNSCCs (**A** and **B**) No difference in cell survival after irradiation or PARPi (1 μM for 2 h) alone in HR-proficient and HR-deficient HNSCCs. (**C** and S1C) Successful inhibition of PAR formation after PARPi in HNSCCs. (**D** and S1D, **E**) Stronger radiosensitization by PARPi in cell lines deficient in HR with higher enhancement ratios (*p* = 0.05). Statistical analysis of at least three different experiments was performed using Student's *t*-test.

It was previously shown that cells carrying mutations in HR genes are highly sensitive to PARPi alone [5, 6]. Radiosensitization by PARPi seems to depend on the fraction of cells in S-phase, the HR mutation status or the switch to PARP-dependent end joining [4, 11, 17, 18]. To test if HR capacity can predict radiosensitization in HNSCCs without known mutations in HR genes cellular sensitivity to single or combined irradiation and PARPi treatment was analyzed. No difference in survival after single treatments was observed between HR-deficient and HR-proficient cell lines (Figure 2A and 2B) although PARP activity was profoundly decreased in all cell lines analyzed after treatment with PARPi (Figures 2C and S1D). However, after combined treatment the HR-deficient group was more effectively radiosensitized by PARPi (Figures 2D and S2A, S2B), showing higher enhancement ratios at 37% survival, with 1.61 ± 0.06 compared to 1.35 ± 0.09 (*p* = 0.05). No correlation was observed between survival after irradiation and the enhancement ratio.

### Pronounced reduction in replication fork elongation in HR-deficient HNSCCs

It was previously shown, that the radiosensitization by PARPi depends on the fraction of cells in S-phase [17, 18]. A model was proposed that HR together with PARP activity is required for the repair of radiation induced damage at active replication forks [7, 27, 28]. One possible mechanism could be that cells deficient in HR fail to restart replication when PARP is inhibited [29], causing an increase of stalled replication forks which the cell attempts to compensate by increased origin firing. The other possible mechanism could be that an increased number of SSBs left unrepaired in PARP-inhibited cells are converted to DSBs at replication forks, causing a reduction of replication tract length and an increase in S-Phase-specific chromosomal damage, such as radial chromosomes.

**Figure 3:**
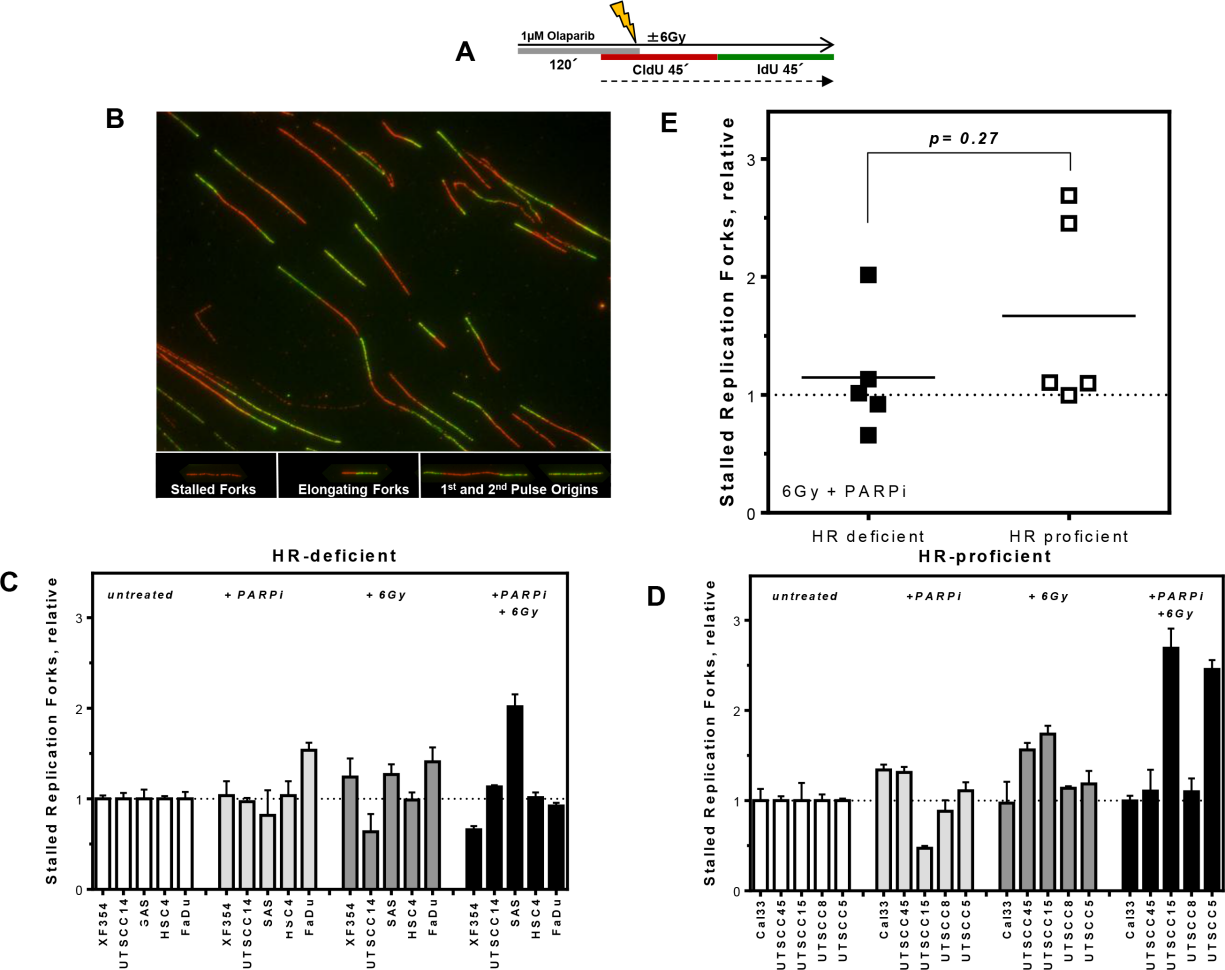
No differences in replication fork stalling after PARPi and irradiation in HR-deficient and HR-proficient HNSCCs (**A**) Labeling protocol for single DNA fiber analysis after treatments and (**B**) representative examples of various types of replication structures. (**C** and **D**) Stalled replication forks in HR-deficient and HR-proficient HNSCCs after PARPi and irradiation alone or after combined treatments as indicated, relative to untreated controls. (**E**) No significant difference in stalled replication forks between both groups (*p* = 0.27). Statistical analysis of at least three different experiments was performed using Student's *t*-test.

**Figure 4:**
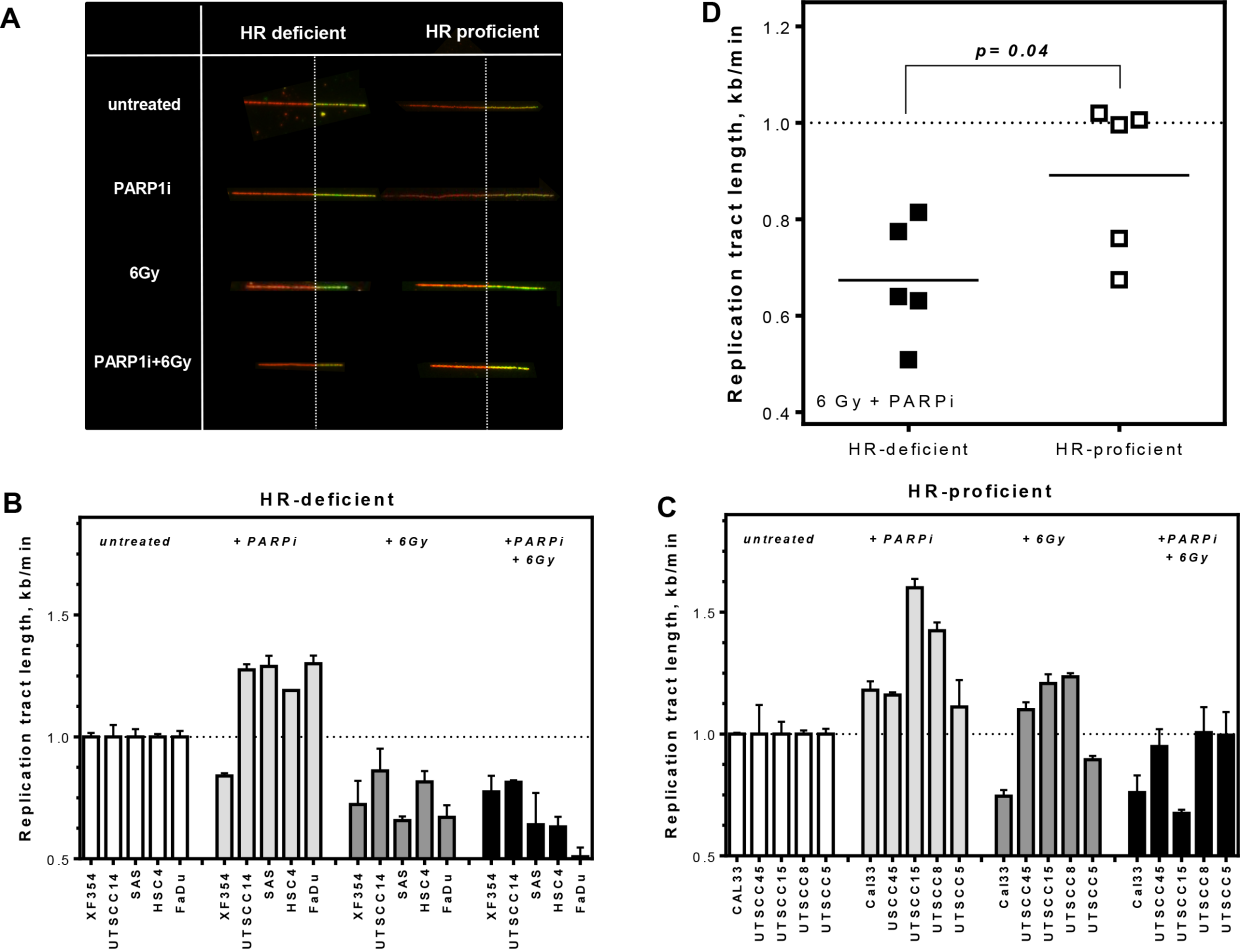
Stronger reduction in replication tract length in HR-deficient compared to HR-proficient HNSCCs after combined treatment (**A**) Examples of replication tracts after different treatments in HR-proficient and -deficient cells. (**B** and **C**) HR-proficient and HR-deficient HNSCCs were sequentially pulse-labeled with CldU followed by IdU and replication tract length (kb/min) was calculated. Replication tract length was plotted for single as well as combined treatment, relatively to untreated controls. (**D**) A stronger reduction in replication tract length was observed in the HR-deficient group (*p* = 0.04). Statistical analysis of at least three different experiments was performed using Student's *t*-test.

To test if the stronger radiosensitizing effect of PARPi in HR-deficient HNSCCs resulted from a failure of DNA repair at replication, replication structures such as stalled replication forks, replication origin firing as well as replication tract length were analyzed (Figures 3, 4 and S2). The extent of replication fork stalling did not differ between HR-deficient and HR-proficient HNSCCs following PARPi, irradiation or combined treatment (*p* = 0.27, Figure 3C–3E). Both groups include cell lines which show pronounced fork stalling. Also, no clear difference in the activity of replication origins was observed between both groups (*p* = 0.26, Figure S2C–S2E).

By contrast, analysis of replication tract length allowed HR-deficient and HR-proficient cell lines to be discriminated after combined treatment with PARPi and irradiation (Figure 4B–4D). Single treatment with PARPi alone provoked longer replication tracts in several HNSCC cell lines independently of HR competence and also irradiation alone caused longer replication tracts in HR-proficient cells compared to untreated controls (Figure 4B and 4C). However, after combined treatment a stronger reduction in replication tract length was observed in HR-deficient compared to HR-proficient cells, with 0.6 vs. 0.95 kb/min, respectively (Figure 4B and 4C, left columns and Figure 4D; *p* = 0.04). This suggests that, although the replication complexes remain stable, indicated by a lack of change in the number of stalled replication forks (Figure 3), HR-deficient cells need longer to remove incoming DNA damage or have less protection against exonuclease activity – both possible reasons for shorter replication tracts after combined treatment.

### Impairment of replication processes leads to an increase in radial chromosomes in HR-deficient HNSCCs

**Figure 5:**
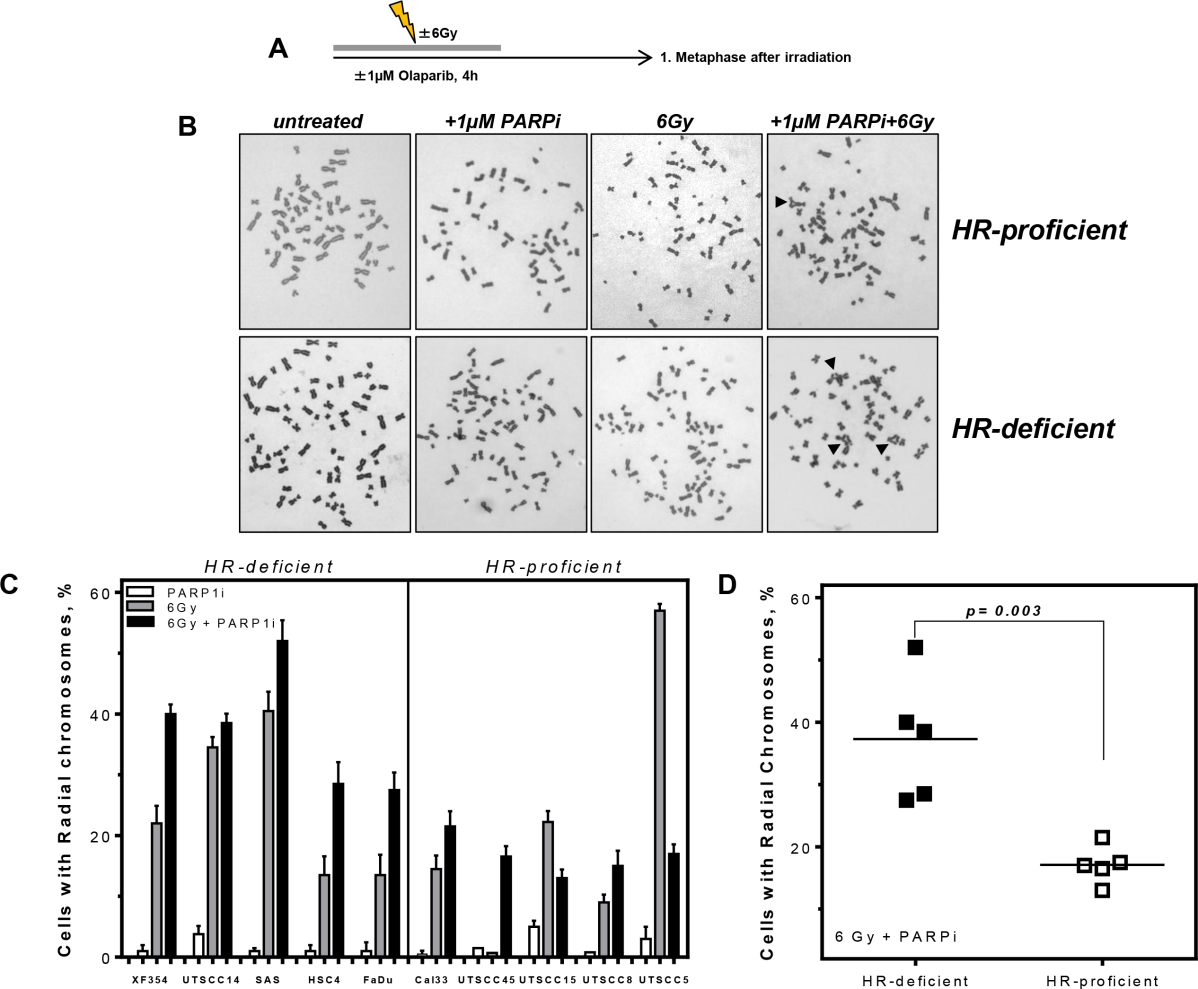
Higher level of radial chromosomes in HR-deficient HNSCC after combined treatment (**A**) HNSCC cells were treated as indicated and 1st metaphases after treatment were analyzed. (**B**) Examples of chromosome preparations show more radial chromosomes (black arrows) in HR-deficient cells after combined treatment of PARPi and irradiation. (**C**) Increase in the percentage of cells with radial chromosomes after PARPi, irradiation and combined treatment in ten HNSCCs. For every cell line analyzed radials were plotted after PARPi, irradiation and combined treatment; untreated controls were subtracted. (**D**) A significantly higher percentage of radial chromosomes after combined treatment was observable in the HR-deficient compared to HR-proficient HNSCCs (*p* = 0.003). Statistical analysis of at least three different experiments was performed using Student's *t*-test.

To further validate the impact of decreased replication fork elongation on cellular survival chromosomal aberrations were measured. Whilst sister chromatid exchanges (SCE) as well as radial chromosomes are known indicators of HR deficiencies [30, 31], SCE do not impact on cellular survival, so we chose radial chromosomes as a read-out of relevance to tumor response. There is no difference in baseline levels of radial chromosomes between HR proficient and deficient cells (data not shown). For every cell line, we plotted the percentage of cells with radials following single and combined treatments after subtraction of untreated controls (Figure 5). HR-deficient HNSCCs showed twice as many cells with radial chromosomes compared to the HR-proficient group after combined treatment with PARPi and 6 Gy X-rays (Figure 5C, 5D) (*p* = 0.003).

Taken together, we show here that HNSCC differ considerably in their HR capacity and that radiosensitization by PARPi depends on HR competence in a replication-dependent manner.

## DISCUSSION

In this study we show that radiosensitization of HNSCC cell lines by PARPi depends on the ability to repair DNA damage via HR at replication forks. Cells with low HR capacity form more radial chromosomes as a consequence of replication failure and are more strongly sensitized to irradiation than cells with high HR capacity.

To our knowledge there are only few publications identifying germline mutations in genes related to DNA repair in HNSCC patients and a direct association to the common HR-deficient phenotype observed in breast and ovarian cancer is missing. However, individuals with the rare and inherited recessive disorder Fanconia anemia (FA) frequently develop HNSCCs [32]. FA proteins repair interstrand-crosslinks, a DNA damage pathway that overlaps functionally with HR during replication. Very recently, FANCO/RAD51C was described as negative prognostic in HNSCCs [33] and other repair factors active at replication forks, like XRCC1 and XPG, are under discussion. Gene expression patterns in HNSCC xenografts after irradiation show a profound up-regulation of CDC45, a regulator of replication initiation and GADD45A, a p53 and BRCA1-regulated stress-inducible gene [34]. Other factors, which might be associated with the observed differences in HR capacity (Figure 1) are mutations in p53, which are present in all cell lines analyzed (data not shown), protein expression of relevant DNA repair factors and cell cycle distribution. Surprisingly, a broad variation in the HR capacity was observed in the ten HNSCC cell lines analyzed, neither reflected by differences in cell cycle nor by expression levels of important proteins. Therefore it seems that minor imbalances in the level or regulation of factors involved in HR are sufficient to influence repair capacity of the whole HR machinery. This can also be observed for breast cancer cell lines [23].

Most of the studies so far have shown that radiosensitization by PARPi was S-phase-dependent. This applies for fibroblasts with a defect in ATM and Artemis [16], as well as human lung, breast, glioma and HNSCC cells [10–12, 17, 18] and also for xenografts of lung cancer and HNSCC [12–14]. However, replication-independent sensitization was observed in Ligase IV deficient fibroblasts [16] and in tumor cell lines of different origins [4]. This was explained by the involvement of the alternative or PARP1-dependent end-joining repair pathway in the radiosensitization process.

However, besides the involvement of PARP1 in DNA repair processes it has a direct role at stalled replication forks in response to replication stress and block MRE11-RAD50-XRS2 resection after fork stalling [9, 35]. Furthermore PAR, supplied by PARP1, interacts directly with Chk1 via a PAR-binding regulatory (PbR) motif in Chk1, allowing an ATR-independent activation of the intra-S phase checkpoint [8]. Our data support the direct role of PARP1 at replication forks. PARPi strongly affected the HR-deficient group by inhibition of the DNA replication elongation response and as a result of failed HR these cells showed twice as many radial chromosomes.

With these data we show that also in HNSCC profound differences exist in the HR capacity and that radiosensitization by inhibition of PARP1 depends on HR competence in a replication-dependent manner. Our observations indicate that PARP inhibitors are promising candidates for enhancing the therapeutic ratio achieved by radiotherapy via disabling DNA replication processes in HR-deficient HNSCCs. However, a powerful tool to select patients with an HR-deficient cancer has to be identified. The analysis of radial chromosomes is technically not feasible in primary biopsies and functional biomarkers in primary tissues are necessary to stratify patients for such a therapy. A promising tool could be the *ex-vivo* detection of RAD51 foci in biopsies derived from tumors to faithfully identify HR-deficient tumors [20].

## MATERIALS AND METHODS

### Cell culture and treatments

Human HNSCC cell lines UTSCC5, UTSCC8, UTSCC14, UTSCC15, UTSCC45, FaDu, Cal33, SAS, HSC4 and XF354 were cultivated in DMEM supplemented with 10% FCS, 2 mM glutamine, 100 U/mL penicillin und 100 μg/mL streptomycin at 37°C at 10% CO_2_. PARPi was achieved by 1 μM olaparib (Selleck^®^), and inhibition of RAD51 by 20 μM RI-1 (Axon Medchem). For the determination of cellular survival cells were treated with up to 1.5 μg/ml mitomycin C (Medac) for 6 h and for irradiation experiments cells were exposed to single radiation doses (2–6 Gy) at a dose rate of 1.2 Gy/min using a Gulmay X-ray machine (GULMAY Medical). All treatments were performed in 37°C and 10% CO_2_ atmosphere.

### Homologous recombination targeting assay

HR capacity was measured by transient transfection of the I-Sce-I-linearized form of the pGC plasmid [36]. 1 μg of linearized plasmid was transfected into cells using FuGENE (Roche) in a 1:3 μg/μl ratio. After 24 h cells were harvested and the fraction of GFP positive cells, representing cells that have successful repaired by HR, was determined by flow cytometry.

### Western blot and immunostaining

Total protein was extracted from exponentially growing cells and 40 μg/ml were resolved by SDS-PAGE using a 4–15% gradient gel (Bio-Rad Laboratories). After transfer proteins were detected by BRCA1 and FANCD2 (Santa Cruz, 1:1000, 1:2000), CHK1 [2G1D5] (Cell Signaling, 1:750), RAD51 [14B4] (1:2.000, GeneTex), PARP1 (BD, 1:1000) or anti-β-actin IgG (1:50.000, Sigma), IRDYE 680 conjugated anti-mouse IgG or IRDYE 800 conjugated anti-rabbit IgG (Licor, 1:7500) or IRDYE 680 conjugated anti-rabbit IgG (Licor, 1:7.500 or 15.000) or IRDYE 800 conjugated anti-mouse IgG (Licor 1:7.500 or 15.000). For chemiluminescence detection ECL^™^ anti-mouse IgG (1:2000, GE Healthcare) and ECL^™^ anti-rabbit IgG (1:2000, GE Healthcare) were used.

For immunofluorescence staining, cells were seeded on culture slides. After treatment as indicated cells were fixed, permeabilized and and blocked overnight. Foci were detected using anti-RAD51 (1:2.000) (Millipore, 1:100), 53BP1 (Novus Biochemicals, 1:250), PAR (Enzo, 1:250) followed by fluoresceinisothiocyanate-linked anti-rabbit IgG (Amersham) or Alexa Fluor 594 goat anti-mouse IgG (Invitrogen) and mounted (Vector Laboratories). Fluorescence images were captured using a Zeiss Axioplan 2 epifluorescence microscope equipped with a charge-coupled device camera and Axiovision software. For quantitative analysis, foci were counted by fluorescence microscopy using a 1,000-fold magnification. 100 cells per dose per slide and experiment were evaluated blindly.

### DNA fiber assay

Exponentially growing cells were pulse labeled with 25 μM CldU (Sigma) followed by 250 μM IdU (Sigma) for 45 min each. For analysis of stalled replication forks after irradiation, olaparib treatment or combined treatment of olaparib and irradiation, CldU and IdU medium was given for 45 min each and DNA damage was induced as indicated during the last 30 min of CldU pulse. Labeled cells were harvested and DNA fiber spreads prepared and stained as described [28]. Fibers were examined using an Axioplan 2 fluorescence microscope (Zeiss). CldU and IdU tracks were measured using ImageJ and micrometer values were converted into kilobases [21, 28]. At least 300 forks were analyzed. Different classes of labeled tracks were classified; red-green (ongoing replication), red (stalled forks) and green (2nd pulse origin). Labeled tracks were counted using ImageJ.

### Metaphase spread analysis

For metaphase spreads exponentially growing cells were treated with colcemid (0.02 μg/ml) overnight, incubated with 0.0075 M KCl, fixed with methanol/acetic acid (3:1), dropped onto microscope slides, stained with 5% giemsa and mounted with entellan before imaging with a Zeiss Axioplan 2 microscope. 100 metaphases per experiment were counted.

### Cell cycle analysis

Cell cycle analysis of exponentially growing cells was performed by flow cytometry (FACS). Cells were fixed, washed with PBS, incubated with propidium iodide (10 μg/ml) RNase (5 μg/ml, Serva) and data were analyzed by flow cytometry using the ModFitLT software on a FACScan (Becton Dickinson) from a cell population under exclusion of debris.

### Clonogenic survival

For survival assays 250 cells were seeded in a 6-well plate 6 h before treatment and cells were cultured for 14 days. Cells were fixed and stained with 1% crystal violet (Sigma-Aldrich, St. Louis, MO). Colonies with more than 50 cells were counted and normalized to untreated samples. Each survival curve represents the mean of at least three independent experiments.

### Statistical analysis

Statistical analysis, curve fitting and graphs were performed using Prism 6.02 (GraphPad Software). Data are given as mean (SE) of 3–5 replicate experiments. Unless stated otherwise, significance was tested by Student's *t*-test.

## SUPPLEMENTARY MATERIALS FIGURES


