# Supplementary material for: PARP1 inhibition radiosensitizes HNSCC cells deficient in homologous recombination by disabling the DNA replication fork elongation response
Source: Oncotarget. 2016 Jan 19;7(9):9732–41. doi: 10.18632/oncotarget.6947 (PMC4891080; doi:10.18632/oncotarget.6947)
Supplement: Supplementary file 1 [file oncotarget-07-09732-s001.pdf]

PARP1 inhibition radiosensitizes HNSCC cells deficient in homologous recombination by disabling the DNA replication fork elongation response

Supplementary Materials

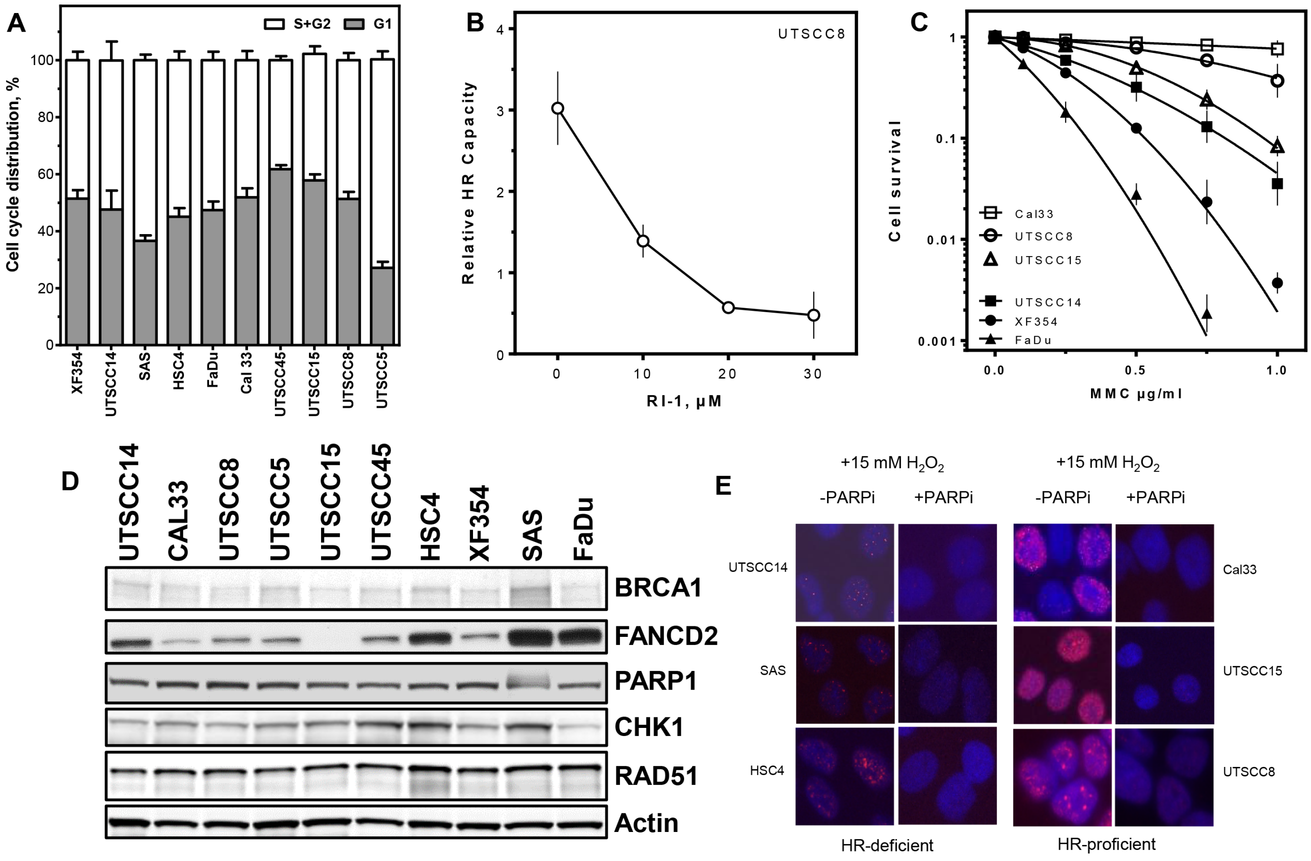

Supplementary Figure S1: Cell cycle distribution, HR capacity, Mitomycin C sensitivity, protein expression pattern and PAR foci formation in ten HNSCCS. (**A**) Cell cycle distributions in ten HNSCC cell lines. Exponentially growing cells were fixed and analyzed by FACS. (**B**) Relative HR capacity in UTSCC8 cells after incubation with increasing RI-1 doses up to 30 µM. (**C**) Cell lines with low HR capacity showed higher sensitivity to mitomycin C compared to cell lines with high HR capacity. Exponential growing cells were treated with increasing doses up to of mitomycin C for 6 h and cellular survival was determined after two weeks. Each curve represents at least three independent experiments. (**D**) Protein expression patterns of BRCA1, FANCD2, PARP1, CHK1 RAD51 and β-actin in ten HNSCC cell lines. (**E**) PAR foci formation in HR-deficient (left) and HR-proficient cell lines (right) after treatment with 15 mM hydrogen peroxide for 20 min.

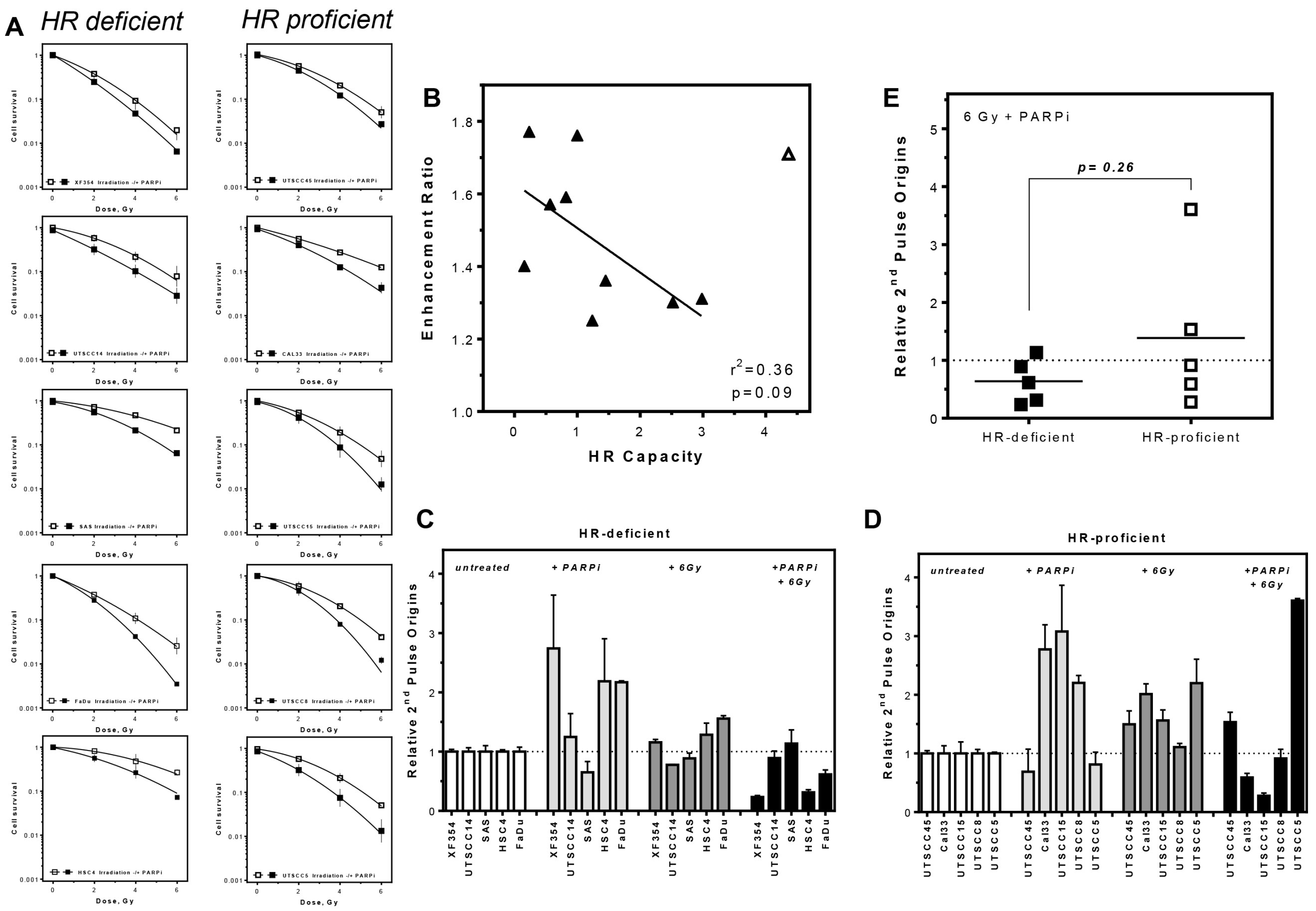

Supplementary Figure S2: PARPi radiosensitizes HR-deficient cells more efficiently than HR-proficient HNSCCs. (**A**) Cell survival after irradiation alone up to 6 Gy and in combination with PARPi. (**B**) Influence of HR capacity on enhancement ratio in HNSCC cell lines. Data were taken from Figure 1A and 2D and fitted by linear regression analysis. (**C**–**E**) Relative frequency of 2nd pulse origin firing in HR-deficient and HR-proficient cell lines. Cells were sequentially pulse-labelled with CldU followed by IdU, origins detected by immunofluorescence with specific antibodies and the percentage of 2nd origins relative to all replication structures was calculated. Statistical analysis of at least three different experiments was performed using Student's *t*-test.
